# Association of Successful Percutaneous Revascularization of Chronic Total Occlusions With Quality of Life: A Systematic Review and Meta-Analysis

**DOI:** 10.1001/jamanetworkopen.2023.24522

**Published:** 2023-07-20

**Authors:** Selcuk Kucukseymen, Mario Iannaccone, James A. Grantham, James Sapontis, Stefan Juricic, Niccolò Ciardetti, Alessio Mattesini, Sinisa Stojkovic, Bradley H. Strauss, Harindra C. Wijeysundera, Gerald S. Werner, Fabrizio D’Ascenzo, Carlo Di Mario

**Affiliations:** 1 Structural Interventional Cardiology, University Hospital Careggi, Florence, Italy; 2 Cardiology Department, San Giovanni Bosco Hospital, ASL Città di Torino, Turin, Italy; 3 Department of Cardiology, Saint Luke’s Mid America Heart Institute, Kansas City, Missouri; 4 Monash Heart, Monash University, Melbourne, Australia; 5 Clinic for Cardiology, University Clinical Center of Serbia, Belgrade, Serbia; 6 School of Medicine, University of Belgrade, Belgrade, Serbia; 7 Schulich Heart Program, Division of Cardiology, University of Toronto, Ontario, Canada; 8 Medizinische Klinik I, Klinikum Darmstadt GmbH, Darmstadt, Germany; 9 Division of Cardiology, Department of Medical Science, University of Turin, Turin, Italy

## Abstract

**Importance:**

Chronic total occlusion percutaneous coronary intervention (CTO-PCI) is not usually offered because of skepticism about long-term clinical benefits.

**Objective:**

To assess the association of successful CTO-PCI with quality of life by analyzing the relevant domains of the Seattle Angina Questionnaire (SAQ).

**Data Sources:**

PubMed, EMBASE, Web of Science, Google Scholar, and Cochrane databases were searched to identify randomized trials and observational studies specifically addressing quality of life domains of SAQ from January 2010 to June 2022.

**Study Selection:**

Studies included reporting SAQ metrics such as angina frequency, physical limitation, and quality of life, before and after CTO-PCI.

**Data Extraction and Synthesis:**

The present study was performed according to the Cochrane Collaboration and Preferred Reporting Items for Systematic Reviews and Meta-Analyses statements, in which fixed-effect or random-effect models with generic inverse-variance weighting depending on statistical homogeneity were applied. Data were extracted by 3 independent reviewers.

**Outcomes and Measures:**

The primary outcome was angina frequency; physical limitation and quality of life were assessed as secondary outcomes.

**Results:**

Seven prospective randomized or observational studies (2500 patients) were included, with a mean (SD) participant age of 61.2 (2.1) years. CTO-PCI was associated with significantly improved quality-of-life metrics during a mean (SD) follow-up of 14.8 (16.3) months. In patients with successful procedures, angina episodes became less frequent (mean [SD] difference for SAQ angina frequency of 12.9 [3.1] survey points [95% CI, 7.1-19.8 survey points]; standardized mean difference was 0.54 [95% CI, 0.21-0.92]; *P* = .002; *I*^2^ = 86.4%) and they experienced less physical activity limitation (mean [SD] difference for SAQ physical limitation of 9.7 [6.2] survey points [95% CI, 3.5-16.2 survey points]; standardized mean difference was 0.42 [95% CI, 0.24-0.55]; *P* < .001; *I*^2^ = 20.9%), and greater quality-of-life domain (mean [SD] difference for SAQ quality of life of 14.9 [3.5] survey points [95% CI, 7.7-22.5 survey points]; standardized mean difference was 0.41 [95% CI, 0.25-0.61]; *P* < .001; *I*^2^ = 58.8%) compared with patients with optimal medical therapy or failed procedure. Furthermore, follow-up duration (point estimate, 0.03; 95% CI, 0.01-0.04; *P* = .01) was associated with a significant decrease in angina frequency in meta-regression analysis.

**Conclusions and Relevance:**

In this systematic review and meta-analysis examining quality of life following CTO-PCI, successful procedures were associated with improved quality-of-life parameters compared with patients on optimal medical therapy or after failed CTO-PCI. These findings suggest support for using PCI to treat CTOs in symptomatic patients unresponsive to medical treatment.

## Key Points

**Question:**

Is successful percutaneous revascularization of chronic total occlusions associated with improved quality of life?

**Findings:**

This meta-analysis of 7 trials, including 2500 patients, found that successful chronic total occlusion revascularization was associated with improved quality of life parameters of patients compared with patients receiving optimal medical therapy or after failed chronic total occlusion revascularization.

**Meaning:**

This study’s results may help physicians in their decision-making process when treating this high-risk group of patients.

## Teaser

This systemic review and meta-analysis investigates whether there is an association of successful chronic total occlusion percutaneous revascularization with quality of life by analyzing the relevant domains of the Seattle Angina Questionnaire.

## Introduction

Chronic total occlusion (CTO) is reported in 15% to 25% of patients with stable coronary artery disease (CAD).^1^ In daily clinical practice, the majority of patients with CTO are treated with optimal medical therapy (OMT), according to the current guidelines, which target (1) angina symptom reduction and (2) prevention of major adverse cardiovascular events (MACE).^2,3,4^ Traditionally, CTO represents 1 of the most challenging lesion subsets in patients undergoing percutaneous coronary intervention (PCI), and CTO-PCI has been associated with low success rates and high complication rates.^5,6^ Despite the advancements in strategies, materials, and the remarkable dedication of experienced operators, the legacy of earlier procedural failures still plagues the dissemination of percutaneous revascularization, with less than 10% of all CTOs being approached with PCI.^3,7^ One of the main obstacles to broader adoption of CTO-PCI is the absence of robust evidence on the benefits of this treatment. Landmark studies in patients with CTO have suggested no difference in mortality between patients treated with PCI compared with OMT, although randomized trials searching for hard outcomes of CTO-PCI are strongly hampered by selection bias and low enrollment.^8,9,10^ Recently, American College of Cardiology/American Heart Association/Society for Cardiovascular Angiography and Interventions 2021 Coronary Revascularization Guideline’s class of recommendation on CTO treatment has been downgraded from IIA to IIB based on 2 randomized trials showing no improvement in left ventricle function following revascularization with a neutral effect on hard outcomes.^11^

There is general agreement that the primary benefit of revascularization in chronic coronary syndromes is symptomatic improvement, convincingly confirmed using the Seattle Angina Questionnaire (SAQ)^12^ in a substudy of the ISCHEMIA trial,^13^ otherwise perceived as a negative trial in terms of hard clinical outcomes for revascularization.^14^ In addition, quality of life scales are generally accepted as critical patient-reported outcome metrics for evaluating interventions in stable CAD.^15,16^ Demonstrating comparable symptom relief and quality of life improvement with percutaneous CTO treatment can support the reappraisal of the class of recommendation and underscore the potential of CTO-PCI revascularization to improve patients’ health status.

A recent review article studied patients’ quality of life (QoL) following CTO revascularization and reported a trend of improvement in QoL indices.^17^ Therefore, we conducted a systematic review and meta-analysis including all available trials assessing the life quality using validated SAQ metrics to test whether successful CTO-PCI is associated with improved quality of life.

## Methods

This meta-analysis study was performed according to the Cochrane Collaboration, Preferred Reporting Items for Systematic Reviews and Meta-analyses (PRISMA) reporting guideline, and the Meta-analysis of Observational Studies in Epidemiology (MOOSE) checklist used for observational studies.^18,19,20^ The study protocol was registered in the PROSPERO database before starting (CRD42022349139).

### Data Sources, Search Strategies, and Data Collection Process

We searched PubMed, EMBASE, Web of Science, Google Scholar, Cochrane Central Register of Controlled Trials, and Cochrane Database of Systematic Reviews from January 2010 to June 2022, using the following search terms separately and in combination: *quality of life*, *Seattle Angina Questionnaire*, *SAQ*, *chronic total occlusion*, *CTO*, *CTO-PCI*, and *CTO revascularization*, to identify published articles reporting on life quality outcomes by using SAQ after CTO-PCI. We also hand-searched bibliographies of identified studies and recent meta-analyses. We restricted our searches to clinical studies, randomized trials, or observational studies. No restrictions were applied regarding language, sample size, or length of follow-up.

### Eligibility and Study Selection Criteria

Publications were selected by predefined criteria and reviewed by 3 cardiologist authors (S.K., C.D.M., M.I.), with differences resolved by consensus. We included published studies that compared the QoL outcomes using SAQ metrics with successful vs failed PCI or OMT alone among patients with CTO. If more than 1 study reported outcomes of the same cohort, we included the most recent or most comprehensive study. We excluded all papers studying non-CTO lesions or treatment with coronary artery bypass grafting (CABG). Patient and lesion characteristics were extracted on a per-protocol analysis, including age, gender, cardiovascular risk factors, SAQ metrics score, and medication. The observational studies identified with this selection process were also assessed for eligibility based on full-text review following the MOOSE checklist^20^ (eAppendix in Supplement 1). Along with data extraction, we evaluated the quality of eligible studies using the Newcastle-Ottawa Scale (NOS),^21^ which contains 8 items classified into 3 domains: selection, comparability, and outcome. The NOS scores ranged from 1 to 9 stars, with 9 stars representing the best quality. Corresponding authors of each study were asked to review the presented data derived from publications.

### Outcomes and Evaluation of Study Quality

The primary outcome was angina frequency (AF) as defined in the SAQ-AF. SAQ physical limitation (SAQ-PL) and SAQ-QoL were assessed as secondary outcomes. Ordinal scores on the questionnaire were transformed to a continuous scale, from a low score of 0 to a maximum score of 100.^22^ The scores of each dimension were analyzed separately. Although variations existed between the follow-up duration, SAQ metrics were reassessed at baseline and follow-up.

The quality of included studies was independently appraised by 2 authors (M.I., F.D.). In addition, for each included study, we evaluated the risk of bias (low, unclear, or high) for random-sequence generation, allocation concealment, blinding of patients and physicians, blinding during the follow-up assessment, preliminary outcome evaluation, and selective reporting, in keeping up with the Cochrane Collaboration approach.

### Statistical Analysis

Continuous variables were reported as mean (SD) or median (IQR). Categorical variables were expressed as number and percentage. Statistical pooling for incidence estimates was performed using a fixed-effect or random-effect model with generic inverse-variance weighting depending on statistical homogeneity, computing risk estimates with 95% CIs using Comprehensive Meta-Analysis software version 3.0 (Biostat Inc). A comparison between successful CTO-PCI, OMT, and failed CTO-PCI groups was performed regarding SAQ-AF, SAQ-PL, and SAQ-QoL in both randomized and observational trials separately. These outcomes were analyzed as continuous variables and the difference between baseline and last follow-up was assessed as standardized mean differences (SMDs). Results were reported as SMDs between groups with 95% CI. In addition, meta-regression analysis was performed to determine the importance of baseline features on the primary outcome and leave-one-out analysis to evaluate any single study effect. Hypothesis testing for statistical homogeneity was set at 2-tailed *P* < .10 and based on the Cochran *Q* test, with heterogeneity score (*I*^2^) values of 25%, 50%, and 75% representing mild, moderate, and severe heterogeneity, respectively.

## Results

### Included Studies and Patient Demographics

**Figure 1.**
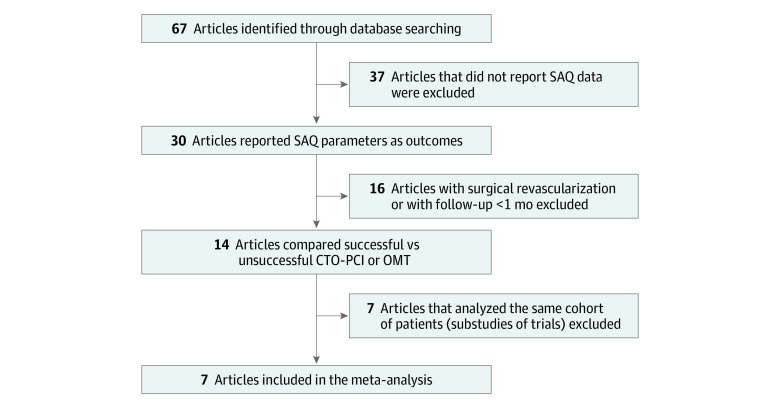
Flow Diagram of Study Selection CTO-PCI indicates chronic total occlusion percutaneous coronary interventions; OMT, optimal medical therapy; SAQ, Seattle Angina Questionnaire.

The flowchart for the search strategy and study inclusion is presented in Figure 1. The initial search strategy found 67 studies eligible for the full-text screening. Among those eligible for full-text screening, 37 were excluded due to a lack of SAQ metrics. We excluded 16 trials that defined revascularization as either PCI or CABG or with a follow-up shorter than 1 month. Seven substudies from included trials were also excluded. Finally, 7 studies^8,22,23,24,25,26,27^ were eligible for inclusion in this meta-analysis, 3 were prospective randomized trials and 4 were prospective observational studies. We used the NOS to evaluate the 7 included studies as described previously. Detailed scores are presented in the eAppendix in Supplement 1. Moreover, the quality scores of these studies are shown in Table 1; the scores were 6 to 9 stars, indicating that the quality of these studies was reliable. They involved 2500 patients with a mean (SD) age of 61.2 (2.1) years; mean (SD) percentage male was 83.2% (89.8%) and mean (SD) percentage female was 16.8% (10.2%). Overall, 35.0% (7.9%) of patients had diabetes, 71.3% (8.5%) had hypertension, 71.6% (3.5%) had dyslipidemia, and 37.7% (10.3%) had current or previous history of smoking. The mean (SD) body mass index (calculated as weight in kilograms divided by height in meters squared) was 27.1 (7.4); the mean (SD) percentage of patients with a history of myocardial infarction was 43.6% (10.0%), and the mean (SD) percentage of patients who had previous CABG was 17.7% (10.7%). Baseline characteristics are summarized in Table 2. The mean (SD) follow-up period was 14.8 (16.3) months.

**Table 1.** Study Design Features and Key Characteristics

Source	Study design	Patients, No. (% of total)	Compared groups	Symptomatic, %	Origin (No. of centers)	Follow-up time, mo	CTO success rate, %	NOS
Grantham et al,^24^ 2010	Prospective observation	125 (5)	Successful vs failed CTO-PCI	65.7	US (1)	1	55	8
Borgia et al,^23^ 2012	Prospective observation	302 (12)	Successful vs failed CTO-PCI	90.8	Europe (1)	48	78	9
Wijeysundera et al,^22^ 2014	Prospective observation	200 (8)	PCI-CTO vs OMT	94	Canada (3)	12	78	9
Sapontis et al,^26^ 2017	Prospective observation	680 (27)	Successful vs failed CTO-PCI	95.8	US (12)	1	86	8
Werner et al,^27^ 2018	Prospective randomized	396 (16)	CTO-PCI vs OMT	100	Europe (28)	12	87	9
Lee et al,^8^ 2019	Prospective randomized	697 (28)	CTO-PCI vs OMT	86.9	Asia (19)	36	91	9
Juricic et al,^25^ 2021	Prospective randomized	100 (4)	CTO-PCI vs OMT	77	Europe (1)	6	94	9

Abbreviations: CTO-PCI, chronic total occlusion percutaneous coronary interventions; NOS, Newcastle-Ottawa Scale; OMT, optimal medical therapy.

**Table 2.** Baseline Demographic Features of Included Patients

Parameters	Patients, mean (SD), %
Total patients, No.	2500
Age, y	61.2 (2.1)
Sex	
Female	16.8 (10.2)
Male	83.2 (89.8)
BMI	27.1 (7.4)
Diabetes	35 (7.9)
Hypertension	71.3 (8.5)
Dyslipidemia	71.6 (3.5)
Smoking	37.7 (10.3)
Prior MI	43.6 (10.0)
Prior CABG	17.7 (10.7)
LAD CTO	27.8 (4.7)
β-blockers	67.5 (18.3)
CCB	22.3 (4.1)
Nitrates	29.7 (13.2)

Abbreviations: BMI, body mass index (calculated as weight in kilograms divided by height in meters squared); CABG, coronary bypass-graft surgery; CCB, calcium channel blockers; CTO, chronic total occlusion; LAD, left anterior descending artery; MI, myocardial infarction.

### Primary Outcome Analysis

**Figure 2.**
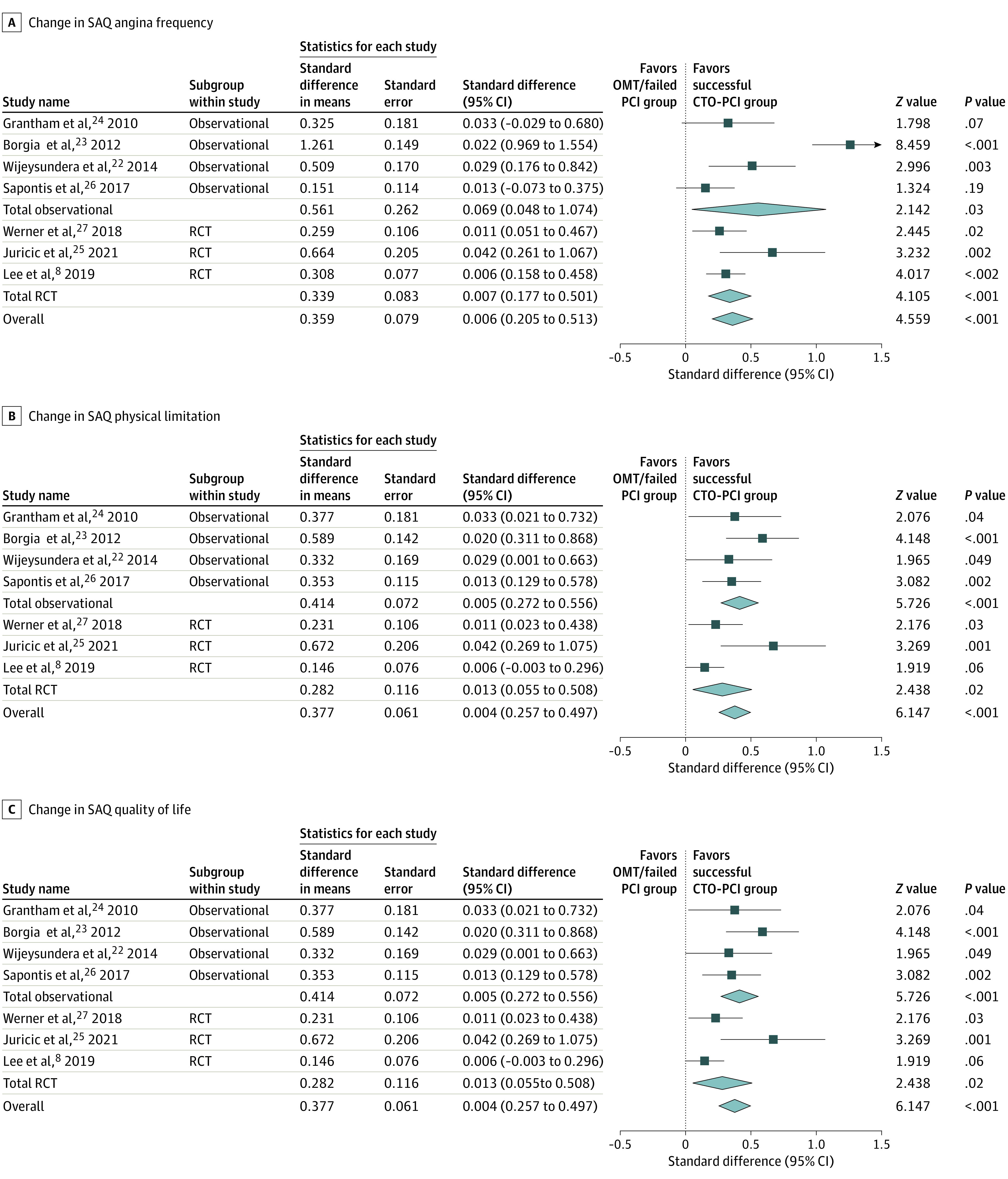
Meta-Analyses Evaluating Association of Successful Chronic Total Occlusion Percutaneous Revascularization (CTO-PCI) Revascularization and Seattle Angina Questionnaire (SAQ) Quality-of-Life Parameters Forest plots show the association of CTO-PCI with SAQ metrics. A, change in SAQ angina frequency. B, Change in SAQ physical limitation. C, Change in SAQ quality of life.

Angina frequency was significantly improved following successful CTO-PCI in 5 out of the 7 contributing studies (2500 participants). The mean (SD) difference for SAQ-AF was 12.9 (3.1) survey points (95% CI, 7.1 to 19.8 survey points) and SMD was 0.54 (95% CI, 0.21 to 0.92; *P* = .002; *I*^2^ = 86.4%) between successful CTO-PCI and combination of failed PCI and OMT. Additionally, meta-regression analysis was performed using the baseline SAQ-AF, which was not found significant (Figure 2A). Also, a longer interval between baseline and final assessment (point estimate, 0.03, 95% CI, 0.01 to 0.04; *P* = .01) was associated with a significant improvement in SAQ-AF. Age, gender, and medications such as calcium canal blockers, β-blockers, and nitrates, did not have an association with results.

### Secondary Outcome Analysis

Results showed a similar favorable association of PL and QoL with the 2 secondary outcome parameters. PL was significantly reduced after successful CTO-PCI revascularization, with a mean (SD) difference of 9.7 (6.2) survey points (95% CI, 3.5 to 16.2 survey points) and SMD of 0.42 (95% CI, 0.24 to 0.55; *P* < .001, *I*^2^ = 20.9%) (Figure 2B). The QoL was reported in 4 out of 6 studies included and was significantly improved after successful CTO-PCI compared with failed PCI and OMT, with a mean (SD) difference of 14.9 (3.5) survey points (95% CI, 7.7 to 22.5 survey points) and SMD of 0.41 (95% CI, 0.25 to 0.61; *P* < .001, *I*^2^ = 58.8%) (Figure 2C).

### Subgroup Analysis of Outcomes

**Figure 3.**
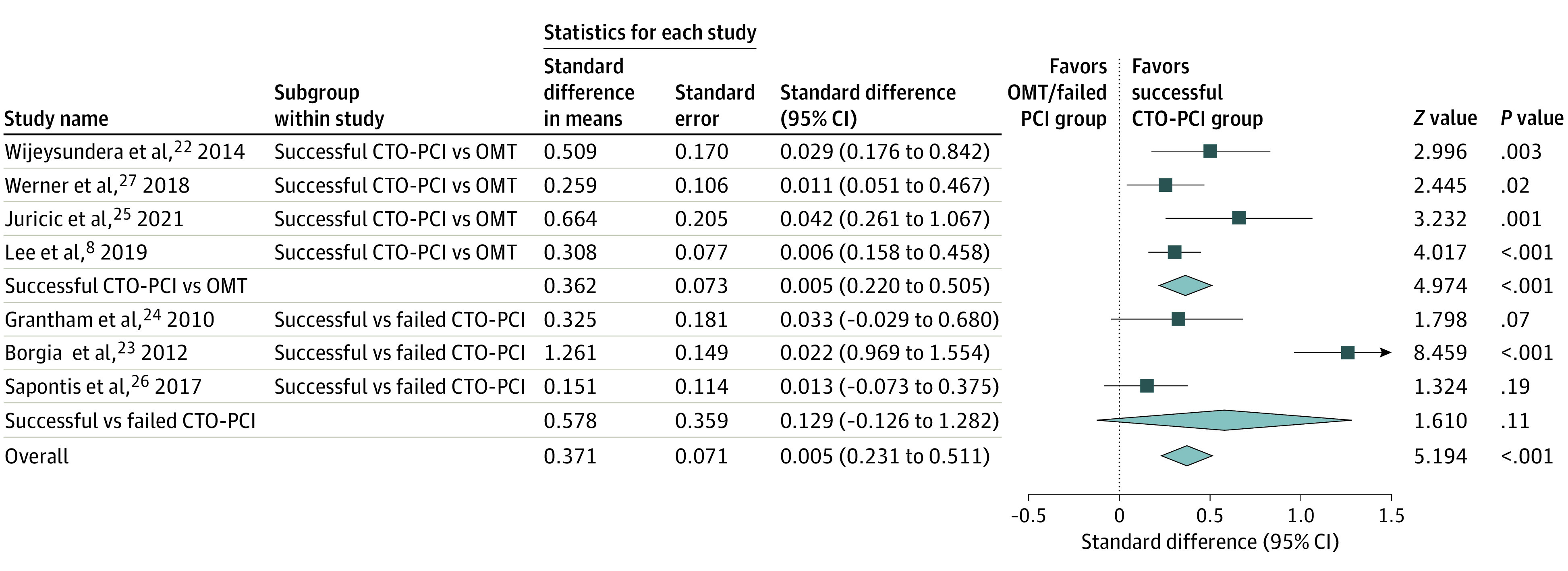
Subgroup Analysis for Each Treatment Group Forest plot shows the association of successful chronic total occlusion percutaneous coronary intervention (CTO-PCI) and the Seattle Angina Questionnaire (SAQ) metrics in subgroup analyses.

Four studies (all 3 randomized, 1 observational) compared successful CTO-PCI with OMT. The other 3 studies compared successful vs failed CTO-PCI. The subgroup analysis for each treatment was performed to further show the association of successful CTO-PCI with the SAQ (Figure 3). As in the primary and secondary outcomes, patients with successful CTO-PCI showed the most improvement in each SAQ metric. Successful CTO-PCI was associated with mean (SD) improvements of 16.1 (5.4) survey points for SAQ-AF, 10.2 (1.5) survey points for SAQ-PL, and 15.8 (3.9) survey points for SAQ-QoL. Moreover, we made indirect comparisons between successful CTO-PCI and each group (successful CTO-PCI vs failed CTO-PCI, and successful CTO-PCI vs OMT), and we found *I*^2^ = 49% for successful PCI-CTO and OMT comparison, and *I*^2^ = 91% for the successful and failed PCI comparison. The pooled main outcome of PCI-CTO and SAQ-AF in RCTs was a risk ratio (RR) of 0.40 (95% CI, 0.05 to 0.85) and in observational data it was an RR of 0.51 (95% CI, 0.07 to 1.15). The pooled main outcome of PCI-CTO and SAQ-PL was an RR of 0.27 (95% CI, 0.13 to 0.55) in RCTs, and in observational data it was an RR of 0.43 (95% CI, 0.38 to 0.59). The pooled main outcome of PCI-CTO and SAQ QoL in RCTs was an RR of 0.43 (95% CI, 0.25 to 0.52), and in observational data it was an RR of 0.41 (95% CI, 0.29 to 0.64). There were no interactions between study designs.

### Publication Bias

The bias evaluation confirmed a high-quality level for all studies included in the primary analysis (eAppendix in Supplement 1). Funnel plot analysis was performed in order to evaluate publication bias in RCTs and observational studies. Visual inspection of funnel plots did not show significant asymmetries, which was validated by the Egger test. The leave-one-out analysis was performed based on quality and to examine the importance of individual studies on the robustness of the primary and secondary outcomes (eAppendix in Supplement 1). There was no change in any outcome following the leave-one-out process.

## Discussion

This meta-analysis included all currently available prospective randomized and observational studies to assess the association of successful CTO-PCI with patient life quality by using subscales of SAQ. Notably, there was an improvement in SAQ scores in patients with CTO who were revascularized successfully. A significant difference developed as early as 30 days after PCI and persisted at the most extended follow-up of 48 months. The success of the CTO-PCI and the severity of symptoms prior to CTO-PCI associated with the symptomatic improvement, expressed as gains in these SAQ metrics.

Life quality changes are often considered soft outcomes compared with the so-called hard end points of death, recurrent myocardial infarction, and new revascularization procedures. Controversially, patient-reported outcomes measures (PROMs) are critical components to assessing whether health care services and practices make a difference to a patient’s life. PROMs are an assessment of health or well-being from the patient’s perspective without interpretation by a clinician or health practitioner collected through patient-reported outcome measures.^28^ The World Health Organization WHO states in its statute, “Health is a state of complete physical, mental and social well-being and not merely the absence of disease or infirmity.”^29^ Therefore, knowledge of a consistent symptomatic improvement following CTO-PCI is essential for physicians when they discuss the potential benefits of this procedure before deciding treatment strategies.^30^

A growing body of evidence indicates benefits, such as symptom improvement and reduced long-term health care costs following CTO-PCI.^31,32^ Small trials managed to use objective measurable changes in exercise performance. The IMPACTOR-CTO trial (94 RCA-CTO patients randomized to CTO-PCI vs OMT alone) found a significant reduction in ischemic burden with an improvement in 6-minute walk distance.^9^ In a study conducted by Sheehy et al,^33^ 78% of the patients were angina-free at 6 months following CTO-PCI. Our study adds to the literature by using the well-validated SAQ questionnaire. To our knowledge, this is the first meta-analysis to formally establish the association of successful CTO-PCI with functional and health status.

Most of the randomized CTO-PCI studies focused on hard end points, and this approach was encouraged by the results of some registries showing a survival benefit.^34,35,36,37^ A different outcome cannot be excluded if a true all-comers trial with sufficiently long-term follow-up is conducted sticking to a strict policy not allowing crossover. In practice, such a trial is unlikely to be feasible and the ethics of a trial forcing half of patients randomized to OMT to live with disabling symptoms for years are questionable. We probably have to accept that, similar to other PCI in other chronic coronary syndromes, the only proven benefit of CTO-PCI is symptom relief. Treatments based on health status–related life quality are therefore becoming more critical in medical decision-making and are recommended by guidelines to aid in treatment strategies for CAD.^15^

The higher procedural and in-hospital complications rate could have determined the worse long-term outcome of patients with failed procedures. This led us to perform a substudy dividing the comparison groups into patients remaining in OMT with no attempt to CTO-PCI and patients with failed CTO-PCI. No differences were observed regarding symptomatic improvement during follow-up, suggesting that the worse outcome was not driven by compromise of collateral flow or other periprocedural complications at the time of the initial CTO-PCI.

It was clearly observed that the quality of life and physical activities were associated with a gradual improvement in patients successfully revascularized with CTO, even in the studies that demonstrated worse or neutral MACE rates.^8,9,10^ As a long-term follow-up study, patients with successful CTO-PCI had an improved physical activity, lesser angina frequency, and greater treatment satisfaction compared with patients with failed CTO-PCI at a 4-year median follow-up.^23^ A large CTO-PCI randomized trial (DECISION-CTO) studied the association of CTO-PCI with life quality^8^ using the SAQ and showed a significant improvement at least at 30 days. Similarly, EURO-CTO trial evaluated life quality metrics among patients whose symptoms were deemed to be genuinely attributed to CTO and showed a consistent significant superiority of CTO-PCI over OMT alone.^27^ Furthermore, there are meta-analyses that have shown successful CTO-PCI is associated with less residual angina in the literature.^17,38,39^ The most recent 1 by Abuzeid et al,^17^ revealed symptomatic improvement following CTO-PCI via either PCI or CABG, and showed improvement in the quality of life. Our current analysis found results in the same direction. However, there are crucial differences: (1) in order to avoid using the same patient group, subgroup analyses were not included, (2) other crucial SAQ metrics were analyzed besides the QoL parameter, (3) only studies that compared successful or unsuccessful CTO-PCI or OMT groups were picked up, and (4) patient groups with CABG treatment were excluded. In this way, we aimed for less margin of error by analyzing more homogeneous patients.

It is important to realize that there is a complex interaction between the CTO vessel and the other coronary arteries through the collateral channels that are present. Therefore, successful results were associated with improved myocardium globally. The EXPLORE trial randomized survivors of a STEMI within 7 days to get PCI or conservative management of a concomitant CTO to observe the alteration in left ventricle ejection fraction at 4 months.^10^ In patients with concurrent CTO in the left anterior descending artery, left ventricle ejection fraction was significantly higher in the CTO-PCI group. Based on cardiovascular physiology, it is not perceived as a surprising finding to see such an improvement following revascularization.^40,41^ It is a phenomenon called collateral (or coronary) steal that appears when the vasodilatory reserve is limited and defined as an absolute or relative fall in coronary blood flow to a vascular region in favor of another supply area under conditions of hyperemia,^42^ which is undoubtedly the case of CTO vessel. The recent study analyzed the flow alternation in the collateral circulation in patients with CTO, before and after successful revascularization.^40^ A significant reduction was shown in collateral flow over time following successful CTO-PCI.

### Limitations

There are some limitations to this study. First, each study carries inherent selection bias invariably associated with patients’ characteristics, CTO site, and time to last SAQ assessment. Second, before-after SMDs have some intrinsic limitations because the baseline and posttest scores are not independent. However, the between-group SMDs (as in our analysis) partially overcame this limitation because they reduced the association of these unmeasurable variables with results at least for the baseline evaluation. Another limitation was that we showed absolute improvement in patients’ life quality using SAQ, instead of using certain thresholds for SAQ domains. The reason was the lack of patient-based data so we could not find patients with AF less than 60. Showing absolute improvement will be correlated much better with long-term outcomes, because some patients with successful CTO-PCI feel better due to physical improvement over time. Therefore, trapping between certain thresholds would limit our long-term estimate. Additionally, the sensitivity analyses and the meta-regression results might have been affected by the small number of the included studies and unmeasured confounding factors and must be interpreted with caution.

## Conclusions

This systematic review and meta-analysis of patients included in 7 randomized trials and registries found a clear improvement in health status–related life quality as assessed by the validated SAQ metrics in patients receiving a successful CTO-PCI. These findings suggest support for using PCI to treat CTOs in symptomatic patients unresponsive to medical treatment.

## Notes

Supplement 1. **eAppendix.** Supplemental Material, Newcastle-Ottawa Scale (NOS), and MOOSE Checklists

Supplement 2.
Data Sharing Statement
