# Supplementary material for: Association of Successful Percutaneous Revascularization of Chronic Total Occlusions With Quality of Life: A Systematic Review and Meta-Analysis
Source: JAMA Netw Open. 2023 Jul 20;6(7):e2324522. doi: 10.1001/jamanetworkopen.2023.24522 (PMC10359963; doi:10.1001/jamanetworkopen.2023.24522)
Supplement: Supplement 1. — eAppendix. Supplemental Material, Newcastle-Ottawa Scale (NOS), and MOOSE Checklists [file jamanetwopen-e2324522-s001.pdf]

Supplemental Online Content

Kucukseymen S, Iannaccone M, Grantham JA, et al. Association of successful percutaneous revascularization of chronic total occlusions with quality of life: a systematic review and meta-analysis. *JAMA Netw Open.* 2023;6(7):e2324522. doi:10.1001/jamanetworkopen.2023.24522

eAppendix. Supplemental Material, Newcastle-Ottawa Scale (NOS), and MOOSE Checklists

This supplemental material has been provided by the authors to give readers additional information about their work.

© 2023 Kucukseymen S et al. *JAMA Network Open.*

eAppendix. Supplemental Material, Newcastle-Ottawa Scale (NOS), and MOOSE Checklists

S1. Supplemental Graphical Abstract

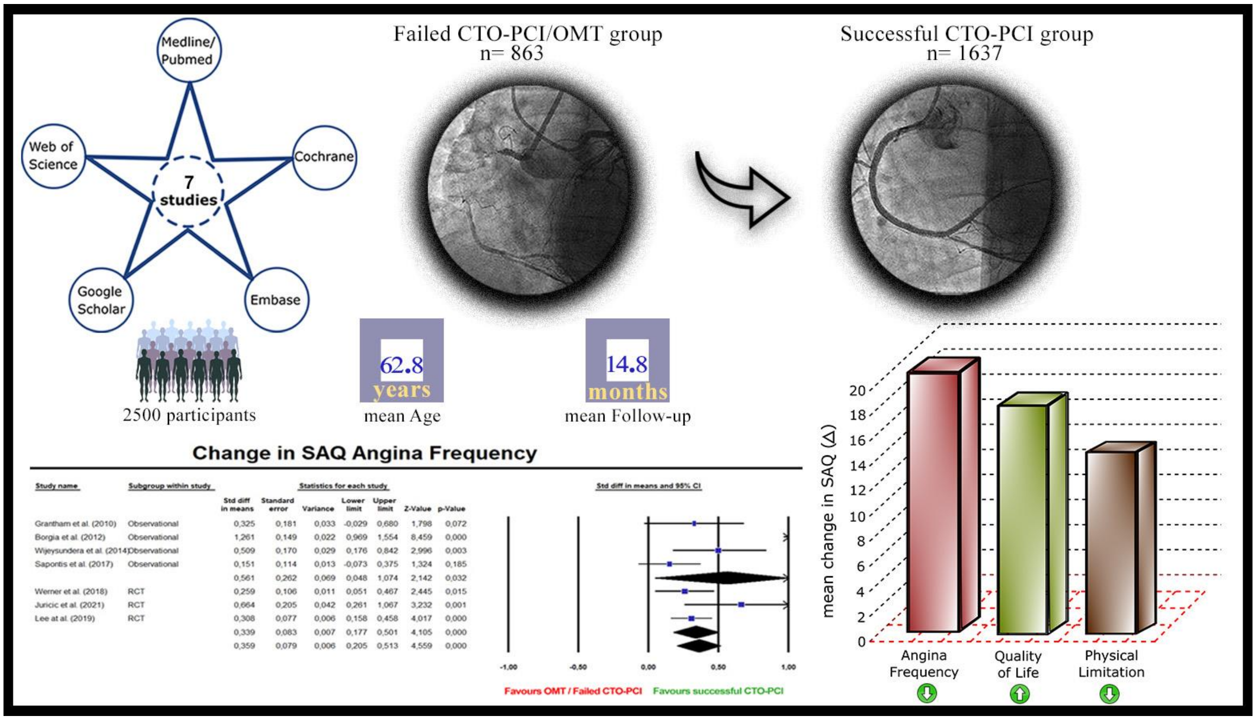

S2. Supplemental results

S2.1. Bias evaluation with Funnel plots

Graphical inspection of funnel plots for each outcome (AF, PL, QoL) did not show significant asymmetry, confirmed by Egger's test. The studies are distributed symmetrically about the combined effect size, suggesting no publication bias.

© 2023 Kucukseymen S et al. *JAMA Network Open.*

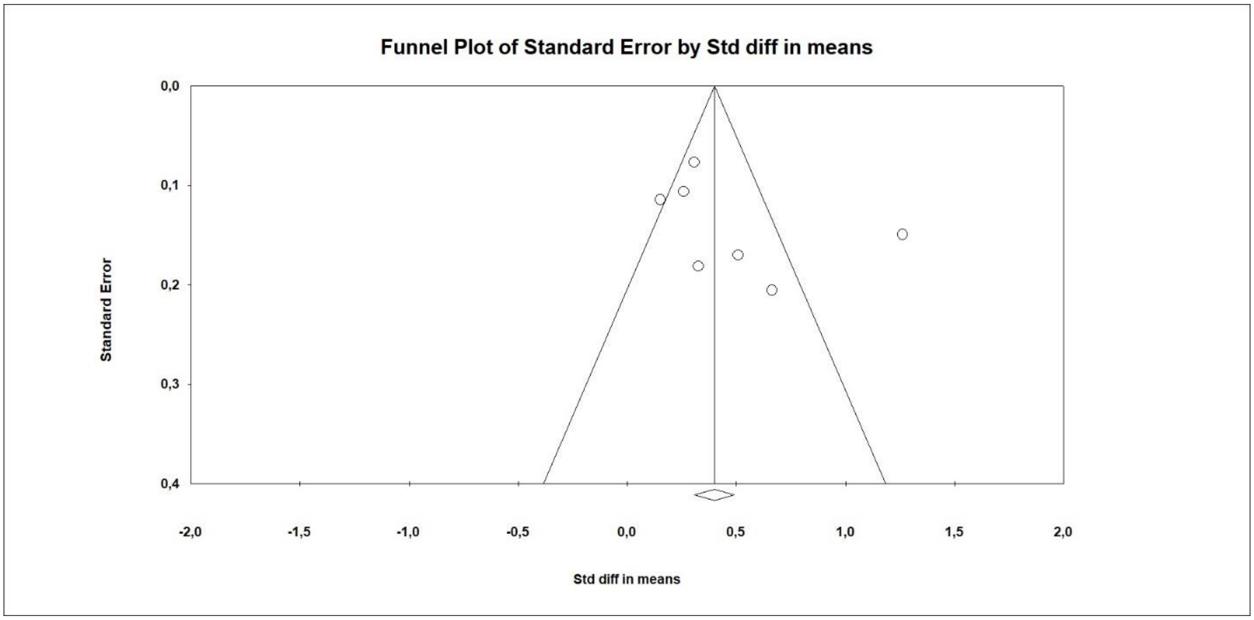

Figure S1. Funnel plot for SAQ Angina frequency metric

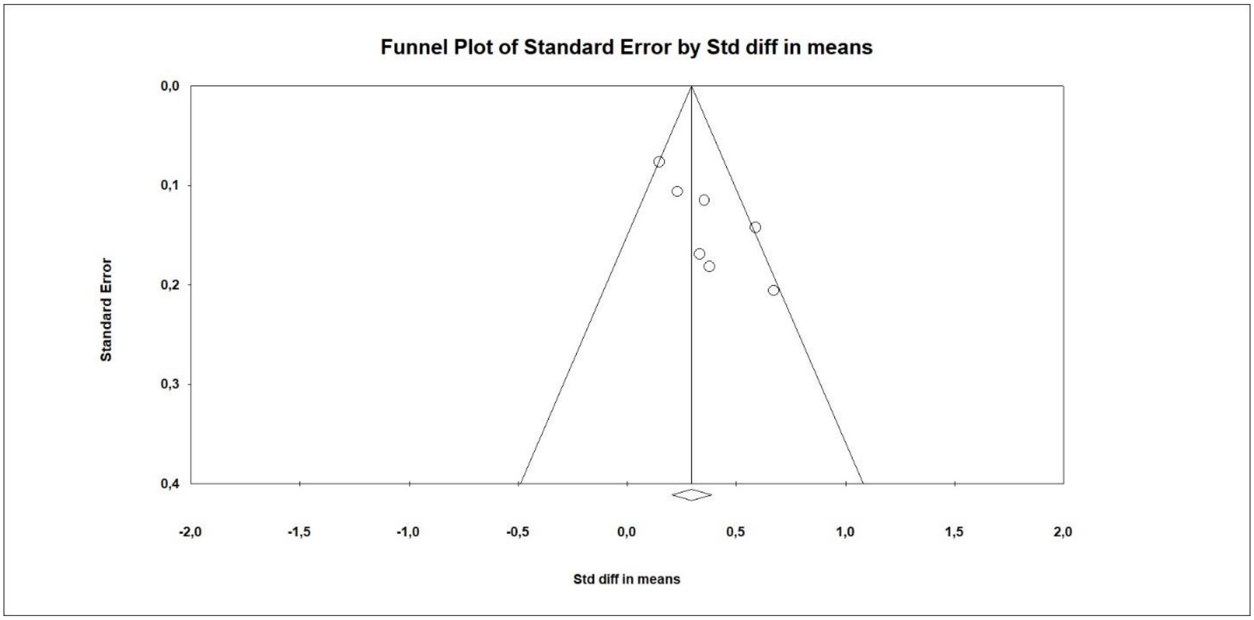

Figure S2. Funnel plot for SAQ Physical limitation metric

© 2023 Kucukseymen S et al. *JAMA Network Open.*

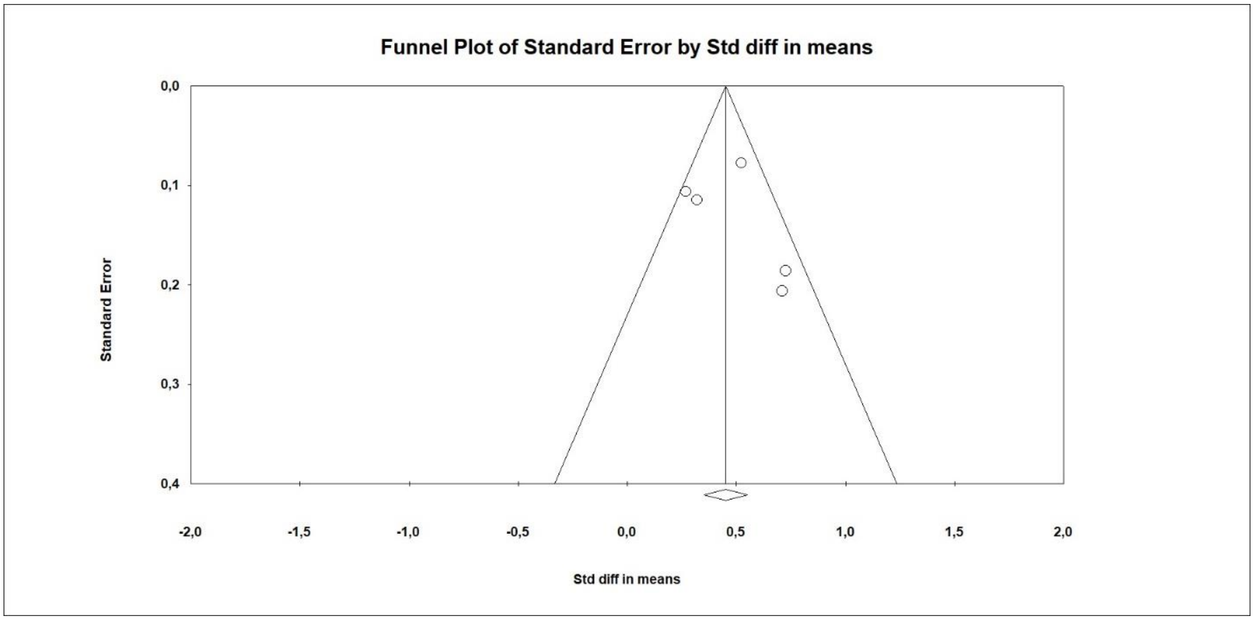

Figure S3. Funnel plot for SAQ Quality of life metric

S1.2. Risk of bias assessment

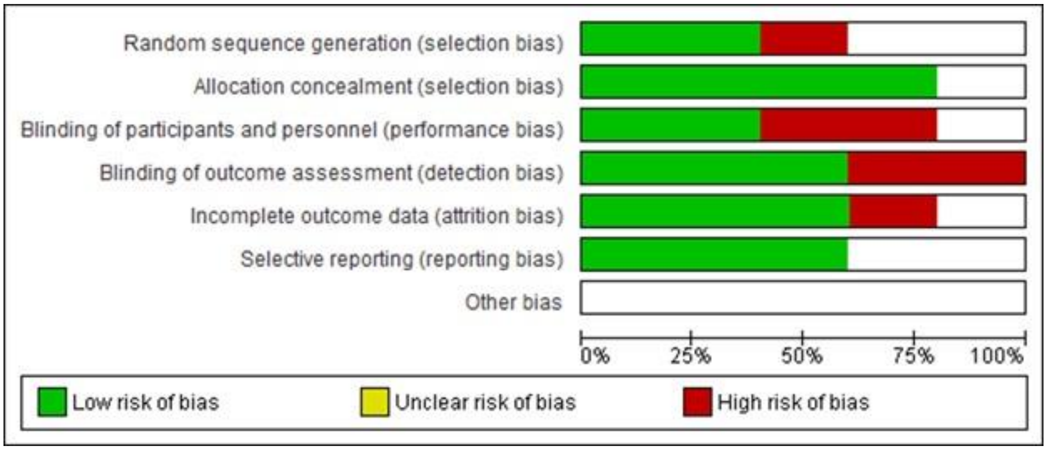

Figure S4. Risk of bias assessment of included trials expressed as low/high/unclear.

© 2023 Kucukseymen S et al. *JAMA Network Open.*

S2.3. Leave-one-out Analyses

Change in SAQ Angina Frequency

Study name	Subgroup within study	Point	Standard error	Variance	Lower limit	Upper limit	Z-Value	p-Value
Grantham et al. (2010)	Successful vs failed CTO PCI	0,403	0,048	0,002	0,310	0,497	8,454	0,000
Borgia et al. (2012)	Successful vs failed CTO PCI	0,307	0,049	0,002	0,212	0,402	6,326	0,000
Wijeysundera et al. (2014)	Successful CTO PCI vs OMT	0,390	0,048	0,002	0,296	0,483	8,125	0,000
Sapontis et al. (2017)	Successful vs failed CTO PCI	0,446	0,050	0,003	0,348	0,545	8,851	0,000
Werner et al. (2018)	Successful CTO PCI vs OMT	0,431	0,051	0,003	0,330	0,531	8,407	0,000
Juricic et al. (2021)	Successful CTO PCI vs OMT	0,384	0,047	0,002	0,291	0,477	8,114	0,000
Lee at al. (2019)	Successful CTO PCI vs OMT	0,450	0,058	0,003	0,337	0,563	7,784	0,000
		0,398	0,046	0,002	0,308	0,489	8,633	0,000

Statistics with study removed

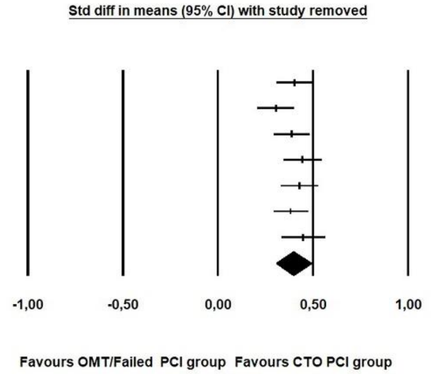

Change in SAQ Physical limitation

Study name	Subgroup within study	Point	Standard error	Variance	Lower limit	Upper limit	Z-Value	p-Value
Grantham et al. (2010)	Observational	0,346	0,080	0,006	0,190	0,502	4,343	0,000
Borgia et al. (2012)	Observational	0,292	0,064	0,004	0,166	0,418	4,542	0,000
Wijeysundera et al. (2014)	Observational	0,352	0,081	0,007	0,193	0,511	4,350	0,000
Sapontis et al. (2017)	Observational	0,351	0,086	0,007	0,183	0,519	4,100	0,000
Werner et al. (2018)	RCT	0,377	0,086	0,007	0,208	0,547	4,363	0,000
Juricic et al. (2021)	RCT	0,308	0,067	0,004	0,177	0,438	4,628	0,000
Lee et al (2019)	RCT	0,390	0,066	0,004	0,261	0,520	5,914	0,000
		0,345	0,071	0,005	0,206	0,484	4,857	0,000

Statistics with study removed

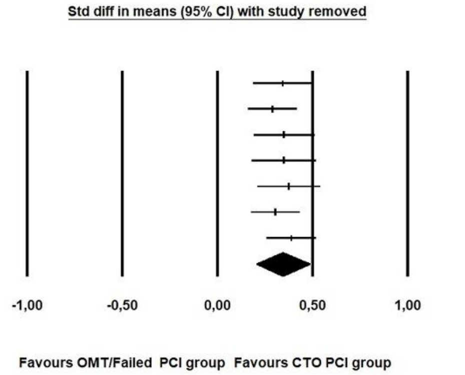

Change in SAQ Quality of Life

Study name	Point	Standard error	Variance	Lower limit	Upper limit	Z-Value	p-Value
Grantham et al. (2010)	0,424	0,085	0,007	0,258	0,590	5,013	0,000
Sapontis et al. (2017)	0,512	0,102	0,010	0,313	0,711	5,037	0,000
Werner et al. (2018)	0,520	0,085	0,007	0,353	0,688	6,100	0,000
Juricic et al. (2021)	0,433	0,088	0,008	0,261	0,605	4,941	0,000
Lee at al (2019)	0,455	0,113	0,013	0,233	0,678	4,012	0,000
	0,465	0,083	0,007	0,302	0,629	5,579	0,000

Statistics with study removed

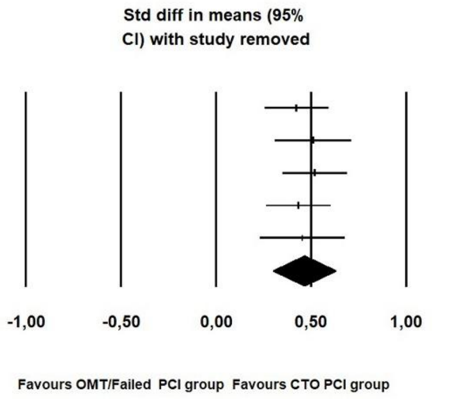

Figure S5. A leave-one-out analysis was performed to examine the impact of individual studies on the robustness of the primary and secondary outcomes. No single study effect at leave one out analysis for all the outcomes (p>0.05).

S2.4. Meta-regression analysis with Log-ratio of events

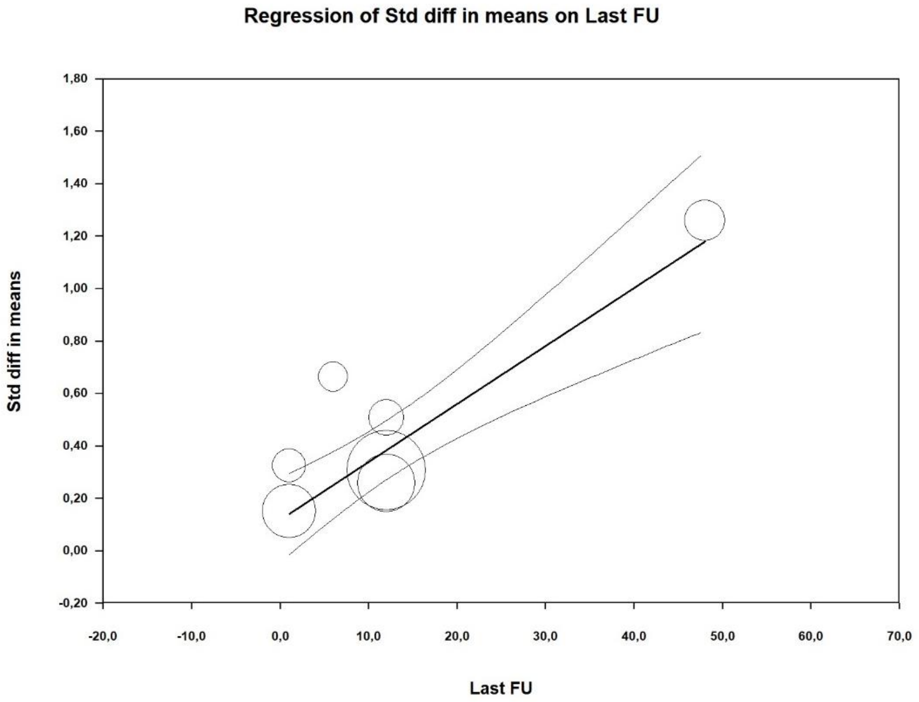

Figure S6. Meta-regression analysis plot describing the effects of follow-up (FU) duration on the proportion of primary outcome.

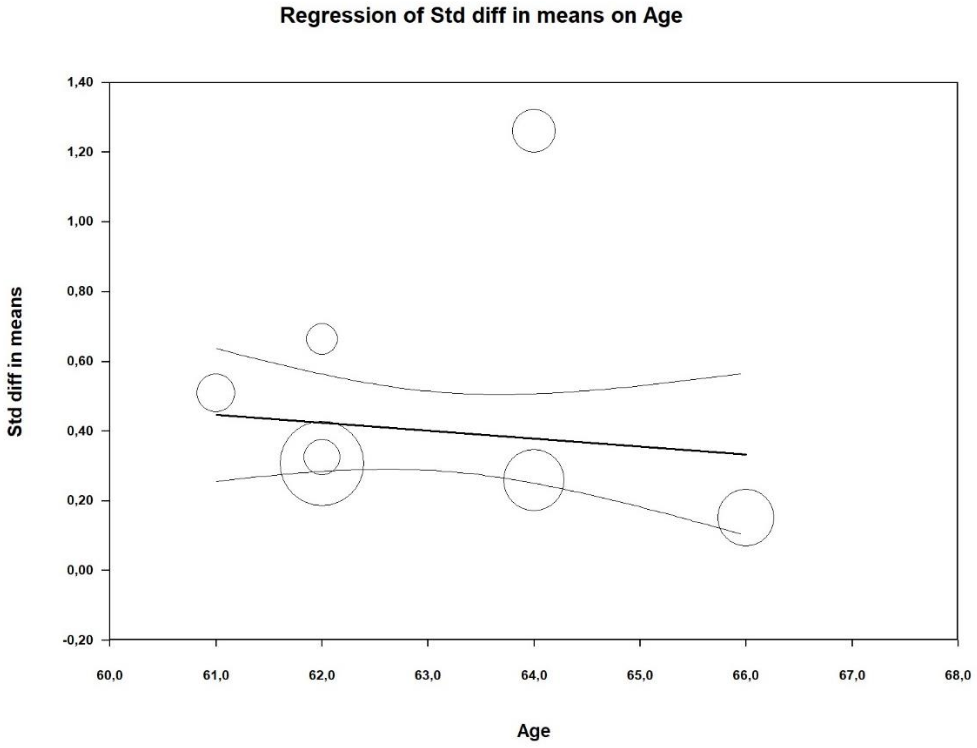

Figure S7. Meta-regression analysis plot describing the effects of age on the proportion of primary outcome.

© 2023 Kucukseymen S et al. *JAMA Network Open.*

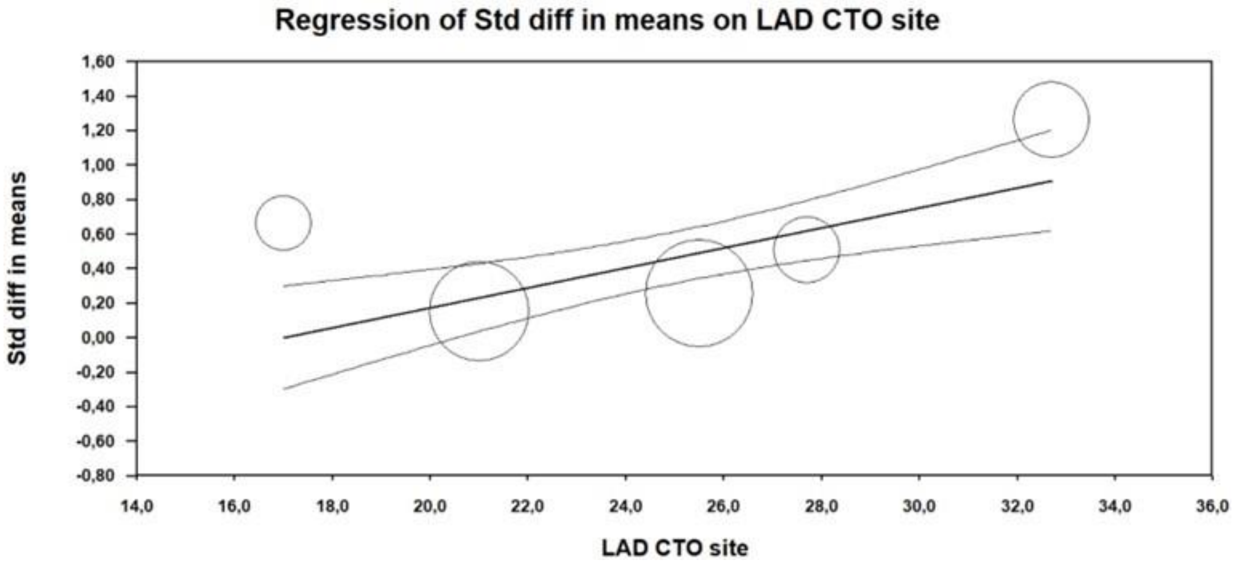

Figure S8. Meta-regression analysis plot describing the effects of successful LAD CTO-PCI on the proportion of primary outcome.

S2.4. Newcastle–Ottawa Scale (NOS)

Studies	Representativeness of the exposed cohort	Selection of non-exposed cohort	Ascertainment of exposure factor	Demonstration that outcome of interest was not present at start of study	Comparability of cohorts on the basis of the design or analysis (★★)	Evaluation of outcome	Was follow-up long enough for outcomes to occur	Adequacy of follow-up of cohorts	Quality Scores
Grantham et al 2010	★	★	★	★	★★	★	★	-	8
Borgia et al 2012	★	★	★	★	★★	★	★	★	9
Wijeysundera et al 2014	★	★	★	★	★★	★	★	★	9
Sapontis et al 2017	★	★	★	★	★★	★	★	-	8
Werner et al 2018	★	★	★	★	★★	★	★	★	9
Lee et al 2019	★	★	★	★	★★	★	★	★	9
Juricic et al 2021	★	★	★	★	★★	★	★	★	9

Notes: "★" represents 1 point, "×" represents 0 point, and "—" represents uncertain points.

© 2023 Kucukseymen S et al. *JAMA Network Open.*
