# Supplementary material for: Association of Successful Percutaneous Revascularization of Chronic Total Occlusions With Quality of Life: A Systematic Review and Meta-Analysis
Source: JAMA Netw Open. 2023 Jul 20;6(7):e2324522. doi: 10.1001/jamanetworkopen.2023.24522 (PMC10359963; doi:10.1001/jamanetworkopen.2023.24522)
Supplement: Supplement 2. — Data Sharing Statement [file jamanetwopen-e2324522-s002.pdf]

Data Sharing Statement

Kucukseymen. Association of Successful Percutaneous Revascularization of Chronic Total Occlusions With Quality of Life. *JAMA Netw Open*. Published July 20, 2023.
doi:10.1001/jamanetworkopen.2023.24522

Data
Data available: Yes
Data types: Deidentified participant data, Data dictionary
How to access data: The studies included to our metaanalysis will be provided.
When available: With publication

Supporting Documents
Document types: None

Additional Information
Who can access the data: List of the studies, and all features of the individuals who were enrolled these studies.
Types of analyses: Statistical
Mechanisms of data availability: Investigator support
